# Diabetic Retinopathy Screening: Predictors and Barriers Among Patients With Diabetes Mellitus in South-West Nigeria

**DOI:** 10.7759/cureus.107755

**Published:** 2026-04-26

**Authors:** Adetunji O Adenekan, Adegboyega S Alabi, Funmi Mayaki, Olubanke T Ilo, Gafar A Yusuf, Olufisayo T Aribaba

**Affiliations:** 1 Ophthalmology, Lagos University Teaching Hospital, Lagos, NGA; 2 Ophthalmology, College of Medicine, University of Lagos, Lagos, NGA; 3 Ophthalmology, General Hospital Ijede, Lagos State Health Service Commission, Lagos, NGA; 4 Ophthalmology, Forever Vision Eye Center, Lagos, NGA

**Keywords:** diabetes mellitus, diabetic retinopathy screening, noncommunicable diseases, patient knowledge, physician referral, retinal examination, screening barriers, screening uptake

## Abstract

Background: Diabetic retinopathy (DR) remains a leading cause of preventable visual impairment globally, particularly in low- and middle-income countries where structured screening programs are limited. This study assessed the uptake of DR screening, identified predictors of attendance, and explored barriers among adults with diabetes mellitus (DM) attending public secondary healthcare facilities in Lagos State, Nigeria.

Methods: A hospital-based cross-sectional study was conducted among 288 adults with confirmed diabetes using a systematic sampling approach. Data were collected using an interviewer-administered questionnaire assessing sociodemographic, clinical, knowledge, and screening characteristics. Retinal examination was performed to determine DR status. Bivariate analyses were conducted using chi-square tests, and multivariate logistic regression identified independent predictors of screening uptake. In-depth interviews with four physicians and eight patients were analyzed thematically to contextualize quantitative findings.

Results: The mean age was 60.4 ± 10.8 years, and 185 (64.2%) were female. Overall, 106 participants (36.8%) reported prior DR screening, while 182 (63.2%) had never been screened. On examination, 85 (29.5%) had DR, including 77 (26.7%) with non-proliferative DR and 3 (1.0%) with proliferative DR. Among never-screened participants, lack of physician referral (166; 91.2%), lack of health insurance (163; 89.6%), and poor awareness (159; 87.4%) were common barriers. Screening uptake was significantly associated with occupation (p = 0.003), duration of diabetes (p = 0.006), previous eye intervention (p < 0.001), good knowledge of DR (p < 0.001), and awareness that diabetes affects the eye (p < 0.001). In multivariate analysis, physician referral was the strongest independent predictor of screening (adjusted odds ratio (AOR) 15.93; 95% CI 4.70-54.07; p < 0.001), followed by good knowledge of DR (AOR 3.73; 95% CI 1.66-8.39; p = 0.002), previous eye intervention (AOR 3.99; 95% CI 1.66-9.57; p = 0.002), and diabetes duration >5 years (AOR 1.94; 95% CI 1.02-3.70; p = 0.043). Qualitative findings revealed symptom-driven referral practices and limited awareness of screening guidelines.

Conclusion: DR screening uptake remains suboptimal in this setting, with provider referral and patient knowledge emerging as critical determinants. Strengthening guideline-based referral systems and structured patient education within diabetes clinics may substantially improve early detection and prevent avoidable blindness.

## Introduction

Diabetes mellitus (DM) represents a major and growing global public health challenge, driven by lifestyle transitions, population growth, and increasing life expectancy [1,2]. Among its microvascular complications, diabetic retinopathy (DR) remains a leading cause of visual impairment and a major contributor to avoidable blindness among people living with diabetes [1]. While DR is frequently cited as a leading cause of vision loss in working-age and older populations, it is more appropriately described as one of the principal causes of preventable vision loss worldwide [3,4]. The risk of developing DR increases with longer duration of diabetes and poorer glycemic control, affecting individuals with both type 1 and type 2 DM [5]. Importantly, evidence suggests that optimal glycemic control alone does not completely eliminate the lifetime risk of DR, underscoring the central role of systematic screening in preventing vision-threatening complications [3].

The burden of DR is substantial across diverse settings. A population-based study in Mexico reported a diabetes prevalence of 21% among individuals aged 50 years and older, with 39% having some degree of DR, 16% diabetic maculopathy, and 8.6% proliferative DR [6]. Similarly, data from hospital-based studies in the United Kingdom indicate that approximately 20% of eyes assessed had proliferative disease, while nearly 10% had center-involving diabetic macular edema [7]. Globally, projections estimate that more than 600 million people will be living with diabetes by 2040, with the majority residing in low- and middle-income countries where health systems often lack the capacity to effectively manage chronic complications such as DR [3].

Effective prevention of diabetes-related blindness relies on timely detection through regular retinal screening, irrespective of the presence or absence of visual symptoms [3,6-9]. The International Council of Ophthalmology (ICO) guidelines recommend a comprehensive dilated eye examination within five years of diagnosis for individuals with type 1 diabetes and at the time of diagnosis for those with type 2 diabetes [3,9,10]. In high-income countries, widespread implementation of screening programs, improved glycemic control, and increased awareness have contributed to declining rates of severe DR [4]. However, similar gains have not been consistently observed in resource-limited settings.

Multiple system-level and patient-level factors influence uptake of DR screening [11]. A similar study from Europe has identified physician referral practices, patient knowledge, comorbidities, age, and access to services as key determinants of screening attendance [12]. In Nigeria, limited awareness of DR and poor understanding of the need for routine eye screening have been identified as major barriers, even among individuals already engaged in diabetes care [13]. National data further highlight a substantial burden of diabetes and DR within the country, reinforcing the need for context-specific strategies to improve service delivery [14]. As diabetes services expand across sub-Saharan Africa and case-finding initiatives for DR are increasingly promoted [5,15], robust local data are required to inform integrated eye-care planning and optimize resource allocation. Understanding the predictors of DR screening uptake alongside patient-reported barriers is essential for designing effective interventions within existing healthcare systems.

This study was designed to address gaps in DR screening within secondary-level care in Nigeria.

The primary objective was to assess the uptake of DR screening and identify independent predictors of screening attendance among adults with DM. The secondary objectives were to (i) examine patient-reported barriers to screening and (ii) explore provider- and patient-level perspectives on referral practices and screening behavior using qualitative methods. These objectives were addressed through a mixed-methods approach integrating quantitative analysis of screening determinants with qualitative insights to contextualize observed patterns.

## Materials and methods

Study design and setting

This was a hospital-based cross-sectional study with a mixed-methods component conducted among adult patients with DM attending medical outpatient clinics of selected public secondary healthcare facilities in Lagos State, Nigeria. The quantitative component assessed uptake of DR screening and associated predictors, while the qualitative component explored provider- and patient-level perspectives on screening practices and barriers.

Study population and eligibility criteria

Eligible participants were consenting adults (≥18 years) with a confirmed diagnosis of DM who had attended the medical outpatient clinic on at least one prior occasion. Patients presenting for their first clinic visit were excluded to ensure prior exposure to routine diabetes care and potential physician counselling regarding DR screening. Patients without confirmed DM were also excluded. Facilities were eligible if they provided routine medical outpatient services for diabetes care but did not offer on-site ophthalmic services, in order to minimize referral and selection bias related to immediate access to eye care. Facilities offering dedicated ophthalmic services were excluded.

Sampling technique and sample size

A systematic sampling technique was employed. The sampling frame consisted of all eligible patients attending the medical outpatient clinics during a two-week study period across selected facilities. Based on average clinic attendance, a sampling interval (k) was determined, and every kth eligible patient was approached after a random starting point was selected each clinic day. Where a selected patient declined participation or did not meet eligibility criteria, the next eligible patient was approached. Recruitment continued until the calculated minimum sample size of 288 participants was reached. The sample size was determined using a single population proportion formula based on prior regional estimates of DR screening uptake, with allowance for non-response.

Data collection and study variables

Data were collected using an interviewer-administered structured questionnaire developed de novo by the investigators specifically for this study, informed by the study objectives, existing literature on DR screening, and the local clinical context. The instrument was designed to capture domains relevant to screening behavior, including sociodemographic characteristics, clinical history, awareness and knowledge of DR, prior screening history, physician referral practices, and perceived barriers to screening. Prior to use, the questionnaire underwent face validation by clinicians involved in diabetes care to ensure contextual relevance and clarity.

The primary outcome variable was prior uptake of DR screening, defined as self-reported history of undergoing retinal examination specifically for DR prior to study enrolment. Good knowledge of DR was defined as a score greater than 50% on the four-item knowledge assessment. Glycemic control was classified based on documented HbA1c values where available, or random blood glucose measurements as recorded in clinic records.

Predictor variables assessed included age, sex, occupation, educational level, duration of diabetes mellitus, glycemic control (based on HbA1c or random blood glucose as documented in clinic records), health insurance status, previous eye intervention, physician recommendation for DR screening, awareness that diabetes affects the eye, and overall knowledge of DR. Among participants without prior screening, perceived barriers evaluated included lack of physician referral, lack of health insurance, poor knowledge or awareness, fear of eye examination, cost of transportation, cost of eye care services, and lack of information regarding screening centers.

Assessment of knowledge of DR

Knowledge of DR was assessed using four core questions evaluating awareness that diabetes affects the eye, awareness that DR can cause blindness, belief that diabetic eye damage is treatable, and understanding that retinal examination is necessary even in the absence of symptoms. Each correct response was assigned one point (maximum score = 4). Scores were converted to percentages; scores ≤50% were classified as poor knowledge, and scores >50% were classified as good knowledge.

Qualitative component

A qualitative component was conducted to contextualize quantitative findings. In-depth interviews were held with four physicians involved in diabetes care and eight adult patients receiving care in participating facilities. Participants were purposively selected to capture diverse experiences. Interviews were audio-recorded, transcribed verbatim, and analyzed using inductive thematic analysis. Codes were generated line-by-line and grouped into broader themes through iterative comparison. Themes explored included referral practices, awareness of DR screening guidelines, symptom-driven care-seeking, and perceived barriers.

Statistical analysis

Quantitative data were analyzed using IBM SPSS Statistics for Windows, Version 23.0 (Released 2015; IBM Corp., Armonk, NY, USA). Continuous variables were summarized using means and standard deviations, while categorical variables were presented as frequencies and percentages. Normality of continuous variables was assessed using the Kolmogorov-Smirnov test. Associations between categorical variables and prior DR screening were examined using Pearson’s chi-square test or Fisher’s exact test where appropriate. Variables with p < 0.20 at bivariate analysis were entered into a multivariate logistic regression model using a backward stepwise approach to identify independent predictors. Adjusted odds ratios (AORs) with 95% CIs were calculated. Statistical significance was set at p ≤ 0.05. There were no missing data for variables included in the final multivariate analysis.

Ethical considerations

Ethical approval was obtained from the Health Research Ethics Committee of Lagos University Teaching Hospital (ADM/DCST/HREC/APP/2878). Administrative approval was also obtained from the Lagos State Health Service Commission and the Medical Directors of participating hospitals. Written informed consent was obtained from all participants. The study adhered to the tenets of the Declaration of Helsinki and was conducted in accordance with good clinical practice guidelines.

## Results

A total of 288 adults with diabetes were included in the analysis. The mean age was 60.4 ± 10.8 years. Females constituted 185 (64.2%) of the cohort, while 103 (35.8%) were male. The majority were of Yoruba ethnicity (245; 85.1%), followed by Igbo (31; 10.8%) and other ethnic groups (12; 4.1%). Christianity was reported by 156 (54.2%) participants and Islam by 132 (45.8%). Most participants were married (211; 73.3%), while 77 (26.7%) were single, separated, or widowed (Table 1).

**Table 1:** Sociodemographic characteristics of participants (n = 288) Descriptive sociodemographic characteristics for the combined study sample (n = 288). Continuous data are reported as mean ± standard deviation; categorical data are counts and column percentages. “Other” ethnicity includes smaller groups recorded in the questionnaire (e.g., Hausa, Edo).

Variable	N	%
Mean age (years), mean ± SD	60.4	± 10.8
Sex		
Female	185	64.2
Male	103	35.8
Ethnicity		
Yoruba	245	85.1
Igbo	31	10.8
Other	12	4.1
Religion		
Christianity	156	54.2
Islam	132	45.8
Marital status		
Married	211	73.3
Single/separated/widow(er)	77	26.7

Regarding socioeconomic characteristics, 167 (58.0%) participants were self-employed, 49 (17.0%) were formally employed, 62 (21.5%) were retired, and 10 (3.5%) were unemployed. Educational attainment was primary level in 94 (32.6%), secondary level in 90 (31.3%), tertiary level in 70 (24.3%), and no formal education in 34 (11.8%). Monthly income was less than ₦20,000 in 173 (60.1%) participants, ₦20,001-50,000 in 51 (17.7%), ₦50,001-100,000 in 41 (14.2%), ₦100,001-500,000 in 21 (7.3%), and greater than ₦500,000 in 2 (0.7%). Health insurance coverage was present in 41 (14.2%), while 247 (85.8%) were uninsured (Table 2).

**Table 2:** Socioeconomic characteristics of participants (n = 288) Categorical socioeconomic variables are presented as counts and column percentages for the combined study sample (n = 288). Income categories are expressed in Nigerian Naira as collected in the original questionnaire; “health insurance” denotes enrolment in any formal health insurance scheme at the time of interview.

Variable	N	%
Employment status		
Employed	49	17.0
Self-employed	167	58.0
Unemployed	10	3.5
Retired	62	21.5
Highest level of education		
None	34	11.8
Primary	94	32.6
Secondary	90	31.3
Tertiary	70	24.3
Monthly income (Naira)		
<20,000	173	60.1
20,001-50,000	51	17.7
50,001-100,000	41	14.2
100,001-500,000	21	7.3
>500,000	2	0.7
Health insurance coverage		
Yes	41	14.2
No	247	85.8

Type 2 diabetes was present in 284 (98.6%) participants and type 1 diabetes in 4 (1.4%). Duration of diabetes was ≤5 years in 180 (62.5%), 6-10 years in 67 (23.3%), and >10 years in 41 (14.2%). Good glycemic control was observed in 203 (70.5%) participants, while 85 (29.5%) had poor control. Previous eye intervention had been performed in 11 (3.8%) participants. Overall, 106 (36.8%) reported prior DR screening, whereas 182 (63.2%) had never undergone screening.

On retinal examination, no DR was identified in 203 (70.5%) participants. Unilateral retinopathy was present in 6 (2.1%), while bilateral disease was observed in 79 (27.4%). Non-proliferative DR was detected in 77 (26.7%), proliferative DR in 3 (1.0%), and advanced diabetic eye disease in 5 (1.7%). Diabetic macular edema was present in 9 (10.6%) of those with retinopathy (n = 85) (Table 3).

**Table 3:** Clinical characteristics of participants (n = 288) This table summarizes the clinical profile of the combined sample of 288 adults with diabetes attending public secondary care clinics. Type and duration of diabetes, glycemic control, prior eye care, prior retinal screening, and findings on clinical retinal examination (DR presence and grade, ADED, and macular edema) are shown.

Variable	N	%
Type of diabetes		
Type 1 DM	4	1.4
Type 2 DM	284	98.6
Duration of diabetes		
≤5 years	180	62.5
6-10 years	67	23.3
>10 years	41	14.2
Mean age at diagnosis (years)		
Type 1	23 ± 1	-
Type 2	55 ± 10	-
Glycemic control (based on HbA1c or RBG)		
Good control	203	70.5
Poor control	85	29.5
Previous eye intervention (any)		
Yes	11	3.8
No	277	96.2
Previous DR screening (self-reported)		
Yes	106	36.8
No	182	63.2
Presence of diabetic retinopathy (clinical exam)		
None	203	70.5
Unilateral DR	6	2.1
Bilateral DR	79	27.4
Type/grade of DR (eye-level grading)		
No DR	203	70.5
Non-proliferative DR (NPDR)	77	26.7
Proliferative DR (PDR)	3	1.0
Advanced diabetic eye disease (ADED)	5	1.7
Diabetic macular edema (DME) (denominator as in source: n = 85)	9	10.6 (of 85)

Awareness that diabetes can affect the eyes was reported by 162 (56.3%) participants. Among these, healthcare workers were cited as the source of information by 95 (58.6%), friends or relatives by 53 (32.7%), and social or mass media by 24 (14.8%). Awareness that diabetes can cause blindness was reported by 160 (55.6%). A total of 155 (53.8%) believed diabetic eye damage can be treated, and 130 (45.1%) believed retinal examination is necessary even in the absence of symptoms. Overall, 149 (51.7%) demonstrated good knowledge of DR, while 139 (48.3%) had poor knowledge (Table 4).

**Table 4:** Knowledge of participants on diabetic retinopathy (n = 288) Knowledge was assessed using four core questions (awareness that DM affects the eye; awareness that DM can cause blindness; belief that diabetic eye damage is treatable; belief that retinal examination is important even in the absence of symptoms). Each correct response = 1 point (maximum score = 4); scores >50% classified as “good” knowledge. “Source of knowledge” allowed multiple responses; percentages shown are % of the 162 participants who reported awareness that DM affects the eye.

Knowledge item	Frequency (n)	Percentage (%)
Knows diabetes can affect the eyes	162	56.3
Source of that knowledge (multiple responses accepted; n = 162)		
Healthcare workers	95	58.6
Friends/relatives	53	32.7
Social media/mass media	24	14.8
Knows diabetes can cause blindness	160	55.6
Believes eye damage from diabetes can be treated	155	53.8
Believes retinal examination is important even when no eye complaints	130	45.1
Has heard specifically of “diabetic retinopathy”	47	16.3
Overall knowledge score		
Good knowledge (score >50%)	149	51.7
Poor knowledge (score ≤50%)	139	48.3

**Figure 1:**
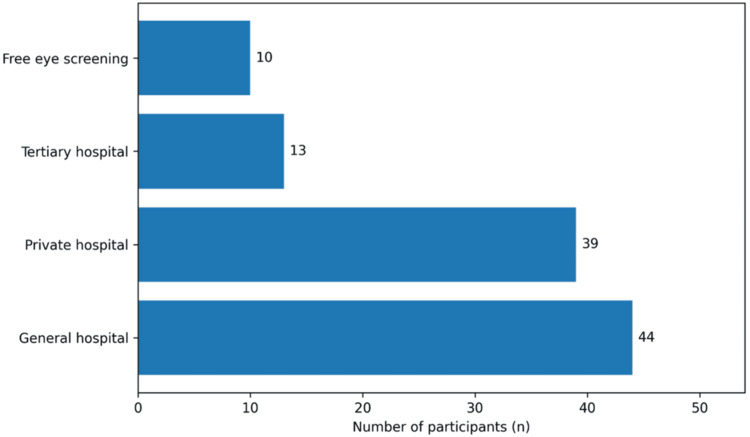
Venue where prior diabetic retinopathy screening was performed among participants who reported a previous retinal examination (n = 106) Free eye screening refers to those who have had eye screening at various free screening programs.

Among the 106 participants who had undergone screening, screening was conducted at general hospitals in 44 (41.5%), private hospitals in 39 (36.8%), tertiary hospitals in 13 (12.3%), and through free eye screening programs in 10 (9.4%) (Figure 1).

**Figure 2:**
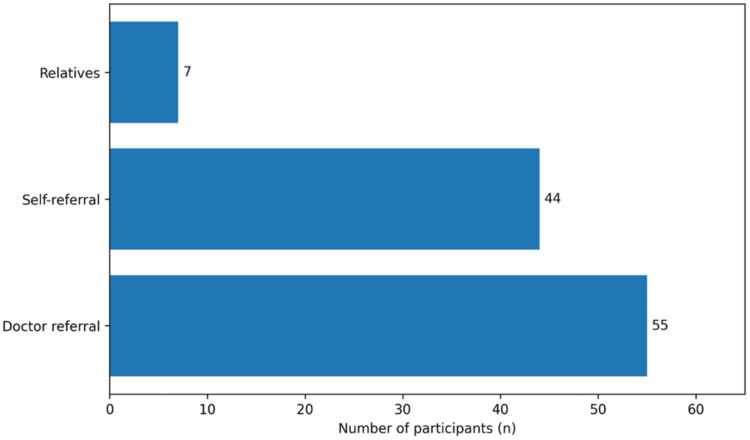
Sources of referral for diabetic retinopathy screening among participants reporting prior retinal examination (n = 106) "Doctor referral” denotes recommendation by a healthcare professional; “self-referral” denotes participants who sought screening without provider prompting; “relatives” denotes referral by family/friends.

Regarding referral pathways among screened participants (n = 106), 55 (51.9%) were referred by healthcare providers, 44 (41.5%) sought screening independently (self-referral), and 7 (6.6%) were referred by relatives (Figure 2).

Among the 182 participants who had never undergone screening, lack of physician referral was reported by 166 (91.2%), lack of health insurance by 163 (89.6%), lack of awareness by 159 (87.4%), and poor knowledge of DR by 105 (57.7%). Fear of eye examination was reported by 33 (18.1%), extra cost of eye care by 9 (4.9%), lack of information about screening centers by 6 (3.3%), and transportation cost by 4 (2.2%) (Table 5).

**Table 5:** Participants’ reported barriers to diabetic retinopathy (DR) screening (n = 182) This table summarizes barriers to diabetic retinopathy screening reported by the 182 participants who had never previously undergone retinal screening. Participants could report more than one barrier; percentages are therefore calculated as the proportion of the 182 never-screened participants.

Barrier reported	Frequency (n)	Percentage of never-screened (n = 182)
Lack of physician referral	166	91.2%
Lack of health insurance	163	89.6%
Poor knowledge of DR/poor awareness	105	57.7%
Lack of patient awareness (general)	159	87.4%
Fear of eye examination	33	18.1%
Extra cost of eye care services	9	4.9%
Lack of information about screening centers	6	3.3%
Cost of transportation	4	2.2%

Bivariate analysis demonstrated no statistically significant association between screening uptake and sex (p = 0.115), religion (p = 0.107), or marital status (p = 0.311). Occupation was significantly associated with screening status (p = 0.003) (Table 6).

**Table 6:** Bivariate association between sociodemographic characteristics and prior diabetic retinopathy (DR) screening (n = 288) This table shows the association between selected sociodemographic characteristics and prior diabetic retinopathy screening among 288 participants. Percentages are column percentages. p-values were calculated using Pearson’s chi-square test. A statistically significant association was observed for occupation (p = 0.003).

Variable	Category	Screened n (%) (n = 106)	Not screened n (%) (n = 182)	χ²	p-value
Sex	Female	62 (58.5)	123 (67.6)	2.41	0.115
	Male	44 (41.5)	59 (32.4)		
Religion	Christianity	51 (48.1)	105 (57.7)	2.48	0.107
	Islam	55 (51.9)	77 (42.3)		
Marital status	Married	74 (69.8)	137 (75.3)	1.02	0.311
	Other (single/separated/widow)	32 (30.2)	45 (24.7)		
Occupation	Employed	24 (22.6)	25 (13.7)	20.27	0.003
	Self-employed	47 (44.3)	120 (65.9)		
	Unemployed	1 (0.9)	9 (4.9)		
	Retired	34 (32.1)	28 (15.4)		

Duration of diabetes was significantly associated with screening uptake (p = 0.006). Previous eye intervention was associated with screening (p < 0.001). Good knowledge of DR was associated with screening (p < 0.001), as was awareness that diabetes affects the eye (p < 0.001). Glycemic control was not significantly associated with screening (p = 0.204) (Table 7).

**Table 7:** Bivariate association between clinical/knowledge characteristics and prior diabetic retinopathy (DR) screening (n = 288) This table presents bivariate associations between selected clinical characteristics and knowledge-related factors and prior diabetic retinopathy screening among 288 participants. Percentages are column percentages. p-values were calculated using Pearson’s chi-square test. Longer duration of diabetes, previous eye intervention, good knowledge of diabetic retinopathy, and awareness that diabetes affects the eye were significantly associated with prior screening uptake.

Variable	Category	Screened n (%) (n = 106)	Not screened n (%) (n = 182)	χ²	p-value
Duration of diabetes	≤5 years	55 (51.9)	125 (68.7)	8.52	0.006
	6-10 years	30 (28.3)	37 (20.3)		
	>10 years	21 (19.8)	20 (11.0)		
Glycemic control	Good	70 (66.0)	133 (73.1)	1.60	0.204
	Poor	36 (34.0)	49 (26.9)		
Previous eye intervention	Yes	10 (9.4)	1 (0.5)	14.39	<0.001
	No	96 (90.6)	181 (99.5)		
Knowledge level	Good knowledge	78 (73.6)	71 (39.0)	32.07	<0.001
	Poor knowledge	28 (26.4)	111 (61.0)		
Awareness that DM affects the eye	Yes	82 (77.4)	80 (44.0)	30.37	<0.001
	No	24 (22.6)	102 (56.0)		

When reported barriers were compared by screening status, lack of physician referral (p < 0.001), lack of health insurance (p < 0.001), poor knowledge (p < 0.001), and fear of examination (p < 0.001) were significantly associated with non-screening. Extra cost of eye care (p = 0.204), lack of information about screening centers (p = 0.233), and transportation cost (p = 0.085) were not statistically significant (Table 8).

**Table 8:** Association between reported barriers and prior diabetic retinopathy (DR) screening (n = 288) This table shows the association between reported barriers and prior diabetic retinopathy screening among 288 participants. Percentages are column percentages. p-values were calculated using Pearson’s chi-square test. Absence of physician referral, lack of health insurance, poor knowledge, and lack of awareness were strongly associated with failure to undergo screening (all p < 0.001). Less frequently reported structural barriers such as transportation cost and information gaps were not statistically significant.

Barrier	Category	Screened n (%)	Not screened n (%)	χ²	p-value
Lack of physician referral	Yes	28 (26.4)	166 (91.2)	127.91	<0.001
	No	78 (73.6)	16 (8.8)		
Lack of health insurance	Yes	40 (37.7)	163 (89.6)	86.48	<0.001
	No	66 (62.3)	19 (10.4)		
Poor knowledge	Yes	17 (16.0)	105 (57.7)	47.60	<0.001
	No	89 (84.0)	77 (42.3)		
Lack of awareness	Yes	24 (22.6)	159 (87.4)	121.12	<0.001
	No	82 (77.4)	23 (12.6)		
Fear of exam	Yes	4 (3.8)	33 (18.1)	12.33	<0.001
	No	102 (96.2)	149 (81.9)		
Extra cost	Yes	2 (1.9)	9 (4.9)	1.71	0.204
	No	104 (98.1)	173 (95.1)		
Lack of info	Yes	1 (0.9)	6 (3.3)	1.56	0.233
	No	105 (99.1)	176 (96.7)		
Transport cost	Yes	0 (0.0)	4 (2.2)	2.36	0.085
	No	106 (100.0)	178 (97.8)		

In multivariate logistic regression analysis, physician referral remained independently associated with screening uptake (AOR 15.93; 95% CI 4.70-54.07; p < 0.001). Good knowledge of DR was independently associated with screening (AOR 3.73; 95% CI 1.66-8.39; p = 0.002), as was previous eye intervention (AOR 3.99; 95% CI 1.66-9.57; p = 0.002). Duration of diabetes greater than five years was associated with screening (AOR 1.94; 95% CI 1.02-3.70; p = 0.043). Health insurance coverage was not statistically significant in the adjusted model (AOR 1.58; 95% CI 0.76-3.29; p = 0.221) (Table 9).

**Table 9:** Multivariate logistic regression showing independent predictors of prior diabetic retinopathy screening (n = 288) This table presents the multivariate logistic regression analysis identifying independent predictors of prior diabetic retinopathy screening among 288 adults with diabetes. Variables entered into the model were those significant at the bivariate level (p < 0.20). Adjusted odds ratios (AOR) with 95% CI are reported. Physician referral emerged as the strongest independent predictor of screening uptake (AOR 15.93; 95% CI 4.70-54.07; p < 0.001). Good knowledge of diabetic retinopathy and previous eye intervention were also independently associated with screening.

Variable	Adjusted odds ratio (AOR)	95% CI	p-value
Physician referral (yes vs no)	15.93	4.70-54.07	<0.001
Good knowledge of DR (yes vs no)	3.73	1.66-8.39	0.002
Previous eye intervention (yes vs no)	3.99	1.66-9.57	0.002
Duration of diabetes (>5 years vs ≤5 years)	1.94	1.02-3.70	0.043
Health insurance (yes vs no)	1.58	0.76-3.29	0.221

Qualitative findings

Participants and Analytic Approach

In-depth interviews were conducted with four physicians involved in diabetes care and eight adult patients with diabetes receiving care in participating public secondary healthcare facilities. Interviews were conducted individually by the principal researcher on separate days. Audio recordings were transcribed verbatim and analyzed using inductive thematic analysis. Initial codes were generated line-by-line and subsequently grouped into higher-order themes through iterative comparison. Linkages between codes were examined using interaction-based analytic techniques, and thematic networks were constructed. Findings are presented without stratification by facility location. Physicians are coded D1-D4 and patients P1-P8.

Physicians’ Perspectives

Limited familiarity with DR screening guidelines: All physicians reported limited knowledge of formal DR screening guidelines. None described the routine use of structured guideline-based referral protocols for patients with diabetes. One physician (D4) stated: “I know that there is a guideline for it. The guideline is related to the stages. I think it is at stage 3 that you refer.” No participant described specific recommendations regarding the timing of the first retinal examination at diagnosis or recommended screening intervals.

Symptom-triggered referral practices: Physicians described referral for retinal evaluation as occurring when patients presented with ocular complaints. Screening discussions were reported to take place primarily in the context of reported visual symptoms. One physician (D2) noted: “Once they have any issue with the eye and they are diabetic, we refer… to the specialist, general hospital or LASUTH.” Another physician (D1) stated: “We don’t refer them unless maybe they come up with a complaint. Based on that we say okay, we need to refer you… Patients are very reluctant about being referred anywhere. If you give them letters they might not go.” Referral destinations mentioned included tertiary hospitals and specialist centers.

Delivery of health education in clinic settings: Physicians reported that health education on diabetic complications, including ocular complications, was conducted within clinic settings, mainly by nurses and dieticians. However, heavy clinic attendance and limited staffing were described. One physician (D2) stated: “With the crowd, there is no time for health talk. Health talk will delay the clinic. They schedule a chosen day for that… but not on the same day of the clinic.” Health talks were described as being scheduled on specific days rather than conducted during routine clinic encounters.

Patients’ Perspectives

Awareness of DR and screening: Among the eight patients interviewed, seven reported that they had never undergone DR screening. Several participants stated that they were unaware that diabetes could affect the eyes. One participant (P7) stated: “It’s only you telling me for the first time.” Another participant (P3) responded: “Diabetic wetin… which word be that? Nobody told me. I didn’t even know it affects the eyes.” Participants did not describe prior exposure to information specifically identifying DR.

Physician referral for screening: Participants reported that retinal screening had not routinely been recommended during clinic visits. Some recalled previous referrals in earlier healthcare settings, while others indicated that no referral had been made.

One participant (P4) stated: “It was once and a long time when I was still attending ENDO clinic… They just asked me to go… I remember I was reluctant… but here… no one said anything like that.” Most participants reported that screening had not been discussed during their current clinic attendance.

Screening in the absence of symptoms: Participants described not seeking screening when they did not experience visual symptoms. One participant (P2) stated: “Because my eyes are not paining me… I can read.” Another participant (P8) stated: “Do I have to go? When I can see and there’s nothing wrong with my eyes…” Screening was discussed by participants in relation to the presence or absence of eye complaints.

## Discussion

This hospital-based cross-sectional study conducted among 288 adults with diabetes in Lagos State provides important contextual evidence on the persistent gap between recommended DR screening practices and real-world uptake in secondary-level public facilities. The overall screening uptake of 36.8% (Table 3) is suboptimal when considered against international recommendations that advocate retinal evaluation at diagnosis for type 2 diabetes and periodic follow-up thereafter [3,9,10]. Although lower coverage rates were reported in structured national programs in high-income countries, it is consistent with patterns described across sub-Saharan Africa, where opportunistic rather than systematic screening predominates [15]. The finding that 63.2% of participants had never undergone screening (Table 3) underscores ongoing vulnerability to preventable visual impairment in a region where DR remains a major cause of avoidable blindness [1,4].

The clinical examination findings reinforce this concern. While 70.5% had no detectable DR, 27.4% had bilateral disease, and 26.7% had non-proliferative DR, with 1.0% demonstrating proliferative DR and 1.7% advanced diabetic eye disease (Table 3). Diabetic macular edema was present in 10.6% of those with retinopathy. These figures align with earlier African epidemiological data demonstrating a substantial burden of sight-threatening disease among individuals with diabetes [1,14,15]. The coexistence of modest screening uptake and measurable advanced pathology suggests delayed case detection, a pattern typical of symptom-driven rather than protocol-driven screening environments [3,4].

Physician referral emerged as the strongest factor independently associated with screening uptake. Among screened participants, 51.9% were referred by healthcare providers (Figure 2), and in multivariate analysis, physician referral was the strongest independent predictor (AOR 15.93; 95% CI 4.70-54.07; p < 0.001) (Table 9). Conversely, 91.2% of never-screened participants cited lack of referral (Table 5), with a strong association at the bivariate level (Table 8). Comparable findings have been reported in Kenya, where provider recommendations significantly predicted uptake [16]. Evidence from Ireland similarly demonstrated improved attendance when screening was delivered through structured primary-care systems [17]. Qualitative work from the United Kingdom further showed that practice-level organization and systematic invitation processes strongly influence screening behavior [18]. Additional qualitative evaluation in UK services confirmed that system-level barriers, rather than deliberate patient avoidance, often underpin non-attendance [19]. The present qualitative findings, highlighting symptom-triggered referrals and limited familiarity with formal DR guidelines among physicians, closely mirror these international observations and indicate weak institutionalization of screening pathways.

Knowledge and awareness constituted the second major driver of screening behavior. Although 56.3% knew diabetes could affect the eyes, only 16.3% had heard specifically of “DR,” and fewer than half recognized the need for examination in the absence of symptoms (Table 4). Good knowledge independently predicted screening (AOR 3.73; p = 0.002) (Table 9), while poor knowledge was strongly associated with non-screening (Table 8). Similar awareness-related barriers have been documented in Nigeria [13] and in Kenya [19]. Dutch primary-care data demonstrated that reminder systems and awareness influence screening attendance [20], while utilization patterns among urban Indonesian patients similarly highlighted knowledge as a determinant of eye care use [21]. Importantly, a randomized controlled trial in rural China demonstrated that structured multimedia education significantly increased uptake of comprehensive eye examination [22]. Evidence from high-risk diabetic populations in the United States also confirms that awareness and provider engagement significantly influence service utilization [23]. Furthermore, program-level data from Oxfordshire indicate that structured organizational systems enhance screening uptake beyond individual-level factors [24]. Qualitative research exploring attitudes and emotional responses to screening similarly shows that anxiety, misconceptions, and service configuration influence attendance decisions [25]. The misconception identified in this study, that screening is unnecessary without visual symptoms, reflects a fundamental misunderstanding of the asymptomatic progression of early DR, a gap repeatedly emphasized in global DR guidance [3,5].

Duration of diabetes greater than five years independently predicted screening uptake (AOR 1.94; p = 0.043) (Table 9), consistent with epidemiological evidence linking longer disease duration to increased retinopathy risk [2,4]. Similar associations between cumulative exposure to diabetes care and the likelihood of retinal examination have been reported in Kenya [16] and Germany [12]. Glycemic control, however, was not significantly associated with screening (Table 7), suggesting that metabolic status alone does not determine preventive eye care behavior.

Socioeconomic vulnerability was marked, with 60.1% earning less than ₦20,000 monthly and 85.8% lacking health insurance (Table 2). Although lack of insurance was strongly associated with non-screening at the bivariate level (Table 8), it did not retain significance in multivariate analysis (Table 9), indicating mediation through referral and knowledge pathways. International evidence suggests that organizational and systemic structures frequently exert greater influence on attendance than direct financial cost [24,25].

Occupation was significantly associated with screening (Table 6), with retirees and formally employed individuals more likely to have undergone retinal examination compared with the self-employed. Similar socioeconomic gradients have been described among hospital-based diabetic populations in the United States [23], underscoring the influence of occupational stability and healthcare engagement patterns.

Finally, the distribution of screening venues (Figure 1) demonstrated reliance on general and private hospitals, with only 9.4% accessing free screening initiatives. This reflects opportunistic service delivery rather than organized population-based screening. In contrast, structured national programs in high-income countries have demonstrated measurable reductions in sight-threatening DR through systematic case identification and recall systems [17].

Collectively, the integrated quantitative and qualitative findings converge on a central conclusion: the primary bottleneck to DR screening in this setting is not simply patient reluctance but weak institutionalization of guideline-driven referral within routine diabetes care. In a region where DR prevalence remains substantial [1,14,15], and where international recommendations clearly define screening timing and intervals [3,9], the prevailing symptom-based approach represents a missed opportunity for blindness prevention. Strengthening provider education, embedding automatic referral triggers within diabetes clinics, and implementing structured patient education strategies may yield greater impact than isolated financial interventions.

This study has several strengths. The mixed-methods design enabled integration of quantitative associations with qualitative insights, thereby strengthening interpretability. The inclusion of clinical retinal examination data, rather than reliance solely on self-report, enhances the validity of the findings. Additionally, the use of multivariate modelling allowed identification of independent predictors of screening uptake.

However, several limitations should be considered. First, the cross-sectional design limits the ability to infer causal relationships between identified predictors and screening behavior. Second, the hospital-based sampling approach may introduce selection bias and limits generalizability to the broader population of individuals with diabetes, particularly those not engaged in routine care. Third, screening history was self-reported and may be subject to recall bias. Fourth, the questionnaire used for this study was developed de novo, based on the study objectives, existing literature, and the local clinical context. It underwent face validation by clinicians involved in diabetes care to ensure clarity and contextual relevance. Formal psychometric validation was not performed. Finally, the qualitative component involved a relatively small number of participants and physicians, and while it provided valuable contextual insights, it may not capture the full diversity of perspectives across settings.

## Conclusions

In conclusion, this study provides comprehensive and contextually grounded evidence on DR screening uptake, predictors, and barriers among adults with diabetes in South-West Nigeria. DR screening uptake in this setting remains suboptimal, with physician referral and patient knowledge emerging as the strongest factors associated with screening attendance. These findings highlight the central role of provider-driven referral practices and patient awareness within routine diabetes care. Strengthening guideline-based referral systems and integrating structured patient education into diabetes services may improve early detection of retinopathy in similar resource-constrained settings.

## References

[1] Burgess PI, MacCormick IJ, Harding SP, Bastawrous A, Beare NA, Garner P (2013). Epidemiology of diabetic retinopathy and maculopathy in Africa: a systematic review. Diabet Med.

[2] Scanlon PH, Aldington SJ, Stratton IM (2013). Epidemiological issues in diabetic retinopathy. Middle East Afr J Ophthalmol.

[3] Wong TY, Sun J, Kawasaki R (2018). Guidelines on Diabetic Eye Care: the International Council of Ophthalmology Recommendations for Screening, Follow-up, Referral, and Treatment Based on Resource Settings. Ophthalmology.

[4] Ting DS, Cheung GC, Wong TY (2016). Diabetic retinopathy: global prevalence, major risk factors, screening practices and public health challenges: a review. Clin Exp Ophthalmol.

[5] von-Bischhoffshausen FB, Castro FM, Gomez-Bastar P (2011). Planning diabetic retinopathy services - lessons from Latin America. Community Eye Health.

[6] Solomon SD, Chew E, Duh EJ (2017). Diabetic retinopathy: a position statement by the American Diabetes Association. Diabetes Care.

[7] Keenan TD, Johnston RL, Donachie PH, Sparrow JM, Stratton IM, Scanlon P (2013). United Kingdom National Ophthalmology Database Study: Diabetic Retinopathy; Report 1: prevalence of centre-involving diabetic macular oedema and other grades of maculopathy and retinopathy in hospital eye services. Eye (Lond).

[8] American Diabetes Association (2017). Standards of medical care in diabetes-2017 abridged for primary care providers. Clin Diabetes.

[9] (2026). International Council of Ophthalmology. ICO guidelines for diabetic eye care. Ophthalmology.

[10] Raman R, Ramasamy K, Shah U (2022). A paradigm shift in the management approaches of proliferative diabetic retinopathy: role of anti-VEGF therapy. Clin Ophthalmol.

[11] Strutton R, Du Chemin A, Stratton IM, Forster AS (2016). System-level and patient-level explanations for non-attendance at diabetic retinopathy screening in Sutton and Merton (London, UK): a qualitative analysis of a service evaluation. BMJ Open.

[12] Kreft D, McGuinness MB, Doblhammer G, Finger RP (2018). Diabetic retinopathy screening in incident diabetes mellitus type 2 in Germany between 2004 and 2013 - a prospective cohort study based on health claims data. PLoS One.

[13] Omolase CO, Olatunde LO, Komolafe OO, Adeleke OE, Akinwalere AK, Omolase BO (2013). Perceptions of barriers to the uptake of diabetic eye screening among diabetic eye patients in Owo, Nigeria. Int J Ophthalmic Pathol.

[14] Kyari F, Tafida A, Sivasubramaniam S, Murthy GV, Peto T, Gilbert CE (2014). Prevalence and risk factors for diabetes and diabetic retinopathy: results from the Nigeria national blindness and visual impairment survey. BMC Public Health.

[15] Burgess PI, Msukwa G, Beare NA (2013). Diabetic retinopathy in sub-Saharan Africa: meeting the challenges of an emerging epidemic. BMC Med.

[16] Mwangi N, Macleod D, Gichuhi S, Muthami L, Moorman C, Bascaran C, Foster A (2017). Predictors of uptake of eye examination in people living with diabetes mellitus in three counties of Kenya. Trop Med Health.

[17] McHugh S, Buckley C, Murphy K (2013). Quality-assured screening for diabetic retinopathy delivered in primary care in Ireland: an observational study. Br J Gen Pract.

[18] Lindenmeyer A, Sturt JA, Hipwell A (2014). Influence of primary care practices on patients' uptake of diabetic retinopathy screening: a qualitative case study. Br J Gen Pract.

[19] Njambi L (2013). Prevalence of diabetic retinopathy and barriers to uptake of diabetic retinopathy screening at Embu Provincial General Hospital, Central Kenya. J Ophthalmol East Cent South Afr.

[20] van Eijk KN, Blom JW, Gussekloo J, Polak BC, Groeneveld Y (2012). Diabetic retinopathy screening in patients with diabetes mellitus in primary care: Incentives and barriers to screening attendance. Diabetes Res Clin Pract.

[21] Adriono G, Wang D, Octavianus C, Congdon N (2011). Use of eye care services among diabetic patients in urban Indonesia. Arch Ophthalmol.

[22] Dan A, Raubvogel G, Chen T (2015). The impact of multimedia education on uptake of comprehensive eye examinations in rural China: a randomized, controlled trial. Ophthalmic Epidemiol.

[23] Maclennan PA, McGwin G Jr, Heckemeyer C (2014). Eye care use among a high-risk diabetic population seen in a public hospital's clinics. JAMA Ophthalmol.

[24] Moreton RB, Stratton IM, Chave SJ, Lipinski H, Scanlon PH (2017). Factors determining uptake of diabetic retinopathy screening in Oxfordshire. Diabet Med.

[25] Hipwell AE, Sturt J, Lindenmeyer A, Stratton I, Gadsby R, O'Hare P, Scanlon PH (2014). Attitudes, access and anguish: a qualitative interview study of staff and patients' experiences of diabetic retinopathy screening. BMJ Open.

